# Medical students’ perceptions, experiences, and barriers towards research implementation at the faculty of medicine, Tanta university

**DOI:** 10.1186/s12909-023-04884-z

**Published:** 2023-11-27

**Authors:** Hisham Ahmed Orebi, Mohamed Reda Shahin, Meret Tawfik Awad Allah, Arwa Hassan Hegazy, Muslim Aqeel Alshakhs, Ahmed Mohamed Alaithan, Abdallah Ahmoud Alhindi, Ibrahim Ali Kabbash

**Affiliations:** 1 Faculty of Medicine, Tanta University, Tanta, Egypt; 2 Professor of Public Health & Community Medicine, Faculty of Medicine, Tanta University, Tanta, Egypt

**Keywords:** Research, Knowledge, Perception, Barriers, Medical students

## Abstract

**Background:**

Research is essential for advancing medical knowledge and improving patient care. However, research capacity and output are low in low- and middle-income countries due to various challenges, including a lack of research training among medical students. Integrating research training into undergraduate medical curricula can help address this issue.

**Methods:**

A cross-sectional study was conducted between December 2022 and March 2023 among 462 undergraduate medical students at Tanta University, Egypt to assess their knowledge, attitudes, and perceived barriers toward conducting research. Data were collected using a self-administered questionnaire and analyzed using SPSS.

**Results:**

Nearly half (49.8%) of the students had an acceptable level of knowledge about research concepts while over two-thirds (66.2%) had a positive attitude. The most common barriers were lack of funding, time, and training in research methods. Previous research training was reported by 66.7% of students, but less than half had participated in or presented research. Students in the competency-based program had significantly higher knowledge and more positive attitudes than those in the mainstream program. Knowledge level was positively correlated with attitude.

**Conclusion:**

While attitudes were generally positive, improvements are needed in research training and opportunities among undergraduate medical students at Tanta University to help address low research capacity challenges in low- and middle-income countries. Integration of formal research training into the curriculum may help increase knowledge and participation in research.

**Supplementary Information:**

The online version contains supplementary material available at 10.1186/s12909-023-04884-z.

## Background

Research is essential in improving health care and plays a central role in the field of medicine. Advances in disease surveillance, diagnosis, treatment, and prevention all rely on the quality of research and the best available evidence. The medical knowledge of the physicians and their training should be up to date, as they are important for their duty in caring for patients and providing the best available effective treatment based on the best available evidence. Additionally, every doctor must contribute to the generation of evidence by conducting research [1].

There are vast differences in the disease burden, research budget allocation, and scientific publications between developed and low- and middle-income countries. There are multiple challenges and opportunities for health research in low- and middle-income countries. One of the primary reasons for reduced research output from low- and middle-income countries is the lack of research capacity [2]. That is why the number of researchers has declined significantly in recent decades, especially in low- and middle-income countries. It is said that health professionals became discouraged about clinical research. A theory points fingers at how the educational system has failed in this area and the lack of programs that stand out as models [3]. According to the literature, several studies suggest that time, financial problems, busy clinical practices, and lack of interest are major obstacles to clinicians’ involvement in research [4, 5].

In the past, undergraduate medical students were not involved much in research because of their vast curriculum, less exposure to research methodology, and lack of time. When these students become postgraduates, their ability to conduct medical research is not up to mark.

The development of research capacity at the undergraduate level is essential to produce high-quality researchers in the long run [6]. Promoting a supportive undergraduate research environment is therefore recommended [7]. Therefore, Medical schools are expected to train undergraduate students to be more familiar with research to support their career prospects and to generate professional researchers [8]. Tomorrow’s clinicians must be equipped with adequate research training during their undergraduate studies to promote their critical thinking, analytical reasoning, communication skills, and application of emerging knowledge to patient care [9].

Most undergraduate medical students are not aware of the importance of research and why it is crucial to health care. Negative attitudes toward research are a major obstacle in learning research associated with poor performance [10]. Attitude, knowledge, and barriers to research are three main factors that have an impact on research success [11]. In Egypt, there is a relatively low knowledge and participation regarding research among undergraduate medical students [12]. For that reason, the aim of conducting this study was to assess the current knowledge and attitudes among medical students at Tanta University in different programs toward conducting research and to identify possible barriers faced by them that might interfere with implementing medical research. The findings of this study will assist in identifying gaps in research training so that programs could be developed to enhance the profile of research in clinical practice and suggest means for improving research training.

## Methods

### Aim of the study

- Assess medical students’ perceptions and attitudes toward conducting research among different medical programs.
- Identify barriers to implementing medical research faced by students and their impact on their perspectives.

### Study design and settings

We carried out a cross-sectional study among undergraduate medical students at the Faculty of Medicine, Tanta University. The study was conducted from December 20th, 2022, to March 20th, 2023. Tanta University enrolls students from Egypt’s governorates in the Nile Delta and has more than 800 medical students at the Faculty of Medicine each year from years 1 to 6, with a population of 5340 students among all medical years.

Medical school at Tanta University offers a 6-year program leading to an MBBCh degree plus a 1-year internship for training to obtain a license to practice. Basic health sciences are the primary focus in the first three years, with gradually increasing exposure to clinical practice over the next three years. Tanta University has recently introduced a new program named (Competency Based Medical Bachelor Program) CBMBP, which is a 6-year medical program also with a 1-year internship but with modified curriculums and new ways of learning, such as problem-based learning (PBL).

### Study participants

The study population was undergraduate medical students at the Faculty of Medicine, Tanta University. The inclusion criteria for this study were all undergraduate medical students at the Faculty of Medicine from year 2 to year 6 from either mainstream or CBMBP. We only excluded 1st -year medical students as they are not yet familiar with research.

The sample size was calculated using Epi Info 7 software created by the Centers for Disease Control and Prevention, Atlanta, Georgia, USA.

The total number of the study population is 5340. With an assumption of a 50% prevalence of good knowledge and attitude among undergraduate medical students based on a literature review of similar studies, an acceptable margin of error of 5%, a level of confidence of 95%, and an 80% power of the study, the calculated sample size was found to be 357. We recruited 462 participants to compensate for any incomplete or invalid questionnaires.

We selected study participants randomly via a cluster random sampling technique by dividing the students in each academic year into small groups according to their attending schedule and randomly selecting 2 groups from each year to collect data from using a self-administered questionnaire.

### Study tool

The authors designed a self-structured questionnaire to collect data based on surveys developed in similar studies conducted in Pakistan [12, 13].

The questionnaire comprised 5 main sections with a total of 52 questions addressing the following:

**Section I**: This section included students’ demographics: age, gender, residence, academic year, and the medical program, which is either the mainstream or the new medical program based on competencies (CBMBP).

#### Section II

This section aimed to assess students’ background information and knowledge about research using 10 multiple-choice questions. We scored right answers by 1 point, whereas wrong answers were assigned 0 points. Thus, a total score of ≥ 7 was designated as acceptable, 5 to < 7 as moderate, and less than 5 as low levels of knowledge.

#### Section III

Students’ attitudes and perceptions toward research using a scale comprised of 19 items. The first 4 items were about students’ perceptions of the term research, and 15 items were about students’ attitudes. The participants were able to express the extent to which they agreed or disagreed with these items using a 5-point Likert scale (strongly agree- agree- neutral- disagree- strongly disagree). We made a scoring system for the 15 attitude questions in the form of the answers given a score of one, two, three, four, or five for the strongly disagree, disagree neutral, agree, and strongly agree or the reverse. A total score of ≤ 30 was assigned as a negative attitude, scores from 31 to 45 were assigned as a neutral attitude, and a total score > 45 was designated as a positive attitude.

#### Section IV

Possible barriers that might interfere with research conduction through 12 items. The participants responded to them using a 5-point Likert Scale (strongly agree- agree- neutral- disagree- strongly disagree).

#### Section V

Students’ previous research experiences or practices through 5 questions, mainly asking about previous research training, previous research involvement, previous research publication, previous research presentation, and if currently working on research. Participants were able to answer these questions by either yes or no.

The questionnaire was reviewed and pilot-tested on 20 undergraduate medical students to check the acceptability and clarity of the questions and the time needed to complete the questionnaire. The pilot responses were not included in the final analysis.

### Statistical analysis

The collected data were organized, tabulated, and statistically analyzed using IBM SPSS version 29 (Statistical Package for Social Studies) created by IBM, Illinois, Chicago, USA. The range, mean, and standard deviation were calculated for numerical values. The number and percentage were calculated for categorical variables and the chi-squared test was used to test differences between subcategories. The level of significance was set at p < 0.05.

### Ethical considerations

The Ethical Committee of Scientific Research in the Tanta Faculty of Medicine approved the research before starting the study. For participants’ consent, we inserted written consent in the introductory page of the questionnaire, and all participants gave informed consent before answering the questions. Participation in this study was voluntary.

## Results

The total number of study participants was 462. Females represented 51.9% of the students, while males represented 48.1%. The mean age of all students was 21.43 ± 1.63 and ranged from 18 to 26 years old. A total of 40.3% of the students were at the preclinical stage, representing the 2nd and 3rd years, while 59.7% of them were in clinical years from the 4th year to the 6th year.

More than half of the students, (57.8%), lived in urban areas. Almost one-quarter of the students (24.9%) were enrolled in the CBMBP (Competency Based Medical Bachelor Program), while 75.1% were mainstream students (Table 1).

The average knowledge score for students was 6.08 ± 2.44 ranging from 0 to 10. Nearly half of the students, 49.8% had an acceptable level of knowledge (≥ 7), 21.6% had a moderate level of knowledge [5, 6], and 28.6% had a low level of knowledge (0–4). (Data not in tables)

**Table 1:** Demographic characteristics of the studied population

Variables	*N* (%) Total *n* = 462
Age	21.43 ± 1.63 (18–26)
Gender	
Male	222 (48.1)
Female	240 (51.9)
Academic grade	
Preclinical	186 (40.3)
Clinical	276 (59.7)
Residence	
Urban	267 (57.8)
Rural	195 (42.2)
Medical Program	
CBMBP	115 (24.9)
Mainstream	347 (75.1)

The average attitude score for the students was 48.55 ± 7.17. Almost two-thirds of the students (66.2%) had a positive attitude toward research with a total attitude score > 45, while 33.1% of them had a neutral attitude with a score ranging from 31 to 45, and only 0.6% had a negative attitude with a total score ≤ 30. (Data not in tables)

When evaluating students’ perception of research, a greater proportion of students 39.2% expressed agreement or strong agreement with the statement “I think I’m aware of research” compared to those who disagreed or strongly disagreed 23.8%. Additionally, more than half of the students 58% agreed or strongly agreed with the idea that research primarily involves testing hypotheses, 60% agreed or strongly agreed that research involves gathering information, and 57.8% agreed or strongly agreed that research involves appraising information. (Table 2)

**Table 2:** Students’ perception towards research

Variables	Perception (Total *N* = 462)		
	Strongly disagree/disagree	Neutral	Agree/ strongly agree
I think I’m aware about research.	110(23.8%)	171 (37.0%)	181(39.2%)
Research is mainly testing hypotheses.	67(14.5%)	127 (27.5%)	268(58.0%)
Research means gathering information.	68 (14.7%)	117 (25.3%)	277(60.0%)
Research means appraising information.	74(16.0%)	121 (26.2%)	267(57.8%)

When evaluating the students’ response rate to potential barriers that could impede research implementation, the most frequently reported barrier was lack of funding, with 35.3% of respondents agreeing and 19.7% strongly agreeing. The second most reported barrier was lack of time, with 35.7% agreeing and 19% strongly agreeing. The third most prevalent barrier was inadequate training in research methods, with 32.7% agreeing and 17.5% strongly agreeing. The remaining barriers were generally more agreed upon. (Table 3)

**Fig. 1:**
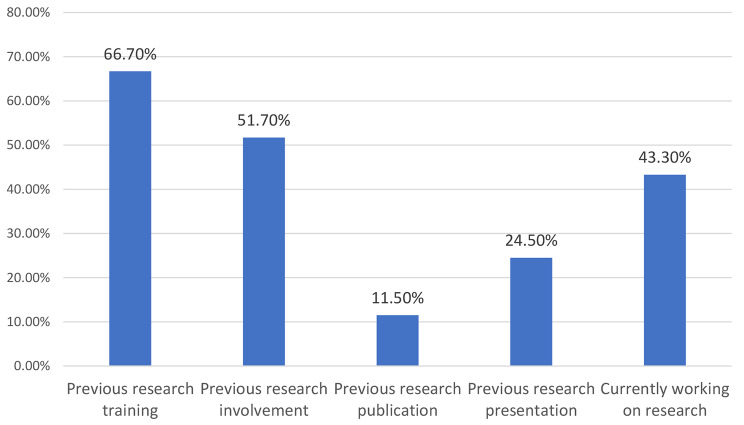
Students’ experiences or practices in research

Regarding students’ prior research experiences or practices, it was found that two-thirds of the students 66.7% had undergone research training, and 51.7% had previously engaged in research activities. However, only 11.5% of them had published research works, and 24.5% had made research presentations. Furthermore, the study revealed that less than half of the students, (43.3%) were actively engaged in research work during the study period. (Fig. 1).

**Table 3:** Medical Students response rate to possible research barriers

Variables	Strongly disagree	Disagree	Neutral	Agree	Strongly agree
Difficulty in following up.	37(8.0%)	71(15.4%)	129 (27.9%)	163(35.3%)	62(13.4%)
Difficulty in obtaining samples.	25(5.4%)	74(16.0%)	144 (31.2%)	164(35.5%)	55(11.9%)
Lack of knowledge	24(5.2%)	108(23.4%)	140 (30.3%)	139(30.1%)	51(11.0%)
Difficulty obtaining approval.	26(5.6%)	76(16.5%)	151 (32.7%)	163(35.3%)	46(10.0%)
Poor accessibility to database.	28(6.1%)	108(23.4%)	132 (28.6%)	144(31.2%)	50(10.8%)
Lack of professional supervisors.	34(7.4%)	116(25.1%)	123 (26.6%)	137(29.7%)	52(11.3%)
Lack of training courses.	36(7.8%)	87(18.8%)	123 (26.6%)	154(33.3%)	62(13.4%)
Lack of time.	20(4.3%)	68(14.7%)	121 (26.2%)	165(35.7%)	88(19.0%)
Lack of funding.	25(5.4%)	58(12.6%)	125 (27.1%)	163(35.3%)	91(19.7%)
Lack of research ideas.	38(8.2%)	114(24.7%)	130 (28.1%)	130(28.1%)	50(10.8%)
Can’t conduct data analysis.	32(6.9%)	107(23.2%)	138 (29.9%)	145(31.4%)	40(8.7%)
No adequate training in research methods.	26(5.6%)	74(16.0%)	130 (28.1%)	151(32.7%)	81(17.5%)

Upon analysis of the student’s level of knowledge about both their gender and academic grade, no significant difference was observed between male and female students, or between preclinical and clinical students. Nonetheless, a significant difference was found when comparing the knowledge level of students from CBMBP and mainstream medical programs, with a significantly higher percentage of CBMBP students (65.2%) exhibiting an acceptable level of knowledge compared to those from mainstream (44.7%). (Table 4)

**Table 4:** Medical students’ level of knowledge in relation to gender, academic grade, and medical program

Variables	**Knowledge level**				X^2^	*P*
	Low (*N* = 132)	Moderate (*N* = 100)	**Acceptable** **(** *N* ** = 230)**	Total (*N* = 462)		
Gender						
Male	**74** 33.3%	**42** 18.9%	**106** 47.7%	**222** 100%	**5.215**	**0.074**
Female	**58** 24.2%	**58** 24.2%	**124** 51.7%	**240** 100%		
Academic grade						
Preclinical	**60** 32.3%	**42** 22.6%	**84** 45.2%	**186** 100%	**2.943**	**0.230**
Clinical	**72** 26.1%	**58** 21.0%	**146** 52.9%	**276** 100%		
Medical Program						
Mainstream	**111** 32.0%	**81** 23.3%	**155** 44.7%	**347** 100%	**14.880**	0.001*
CBMBP	**21** 18.3%	**19** 16.5%	**75** 65.2%	**115** 100%		

When examining the relationship between students’ attitude levels and their gender and academic grades, no significant differences were found between male and female students, or between preclinical and clinical students. Nevertheless, a significant difference was observed when comparing the attitude level of students from CBMBP and mainstream medical programs. The analysis revealed that a significantly higher proportion of CBMBP students (78.3%) exhibited an overall positive attitude toward research, compared to mainstream students (62.2%). (Table 5)

**Fig. 2:**
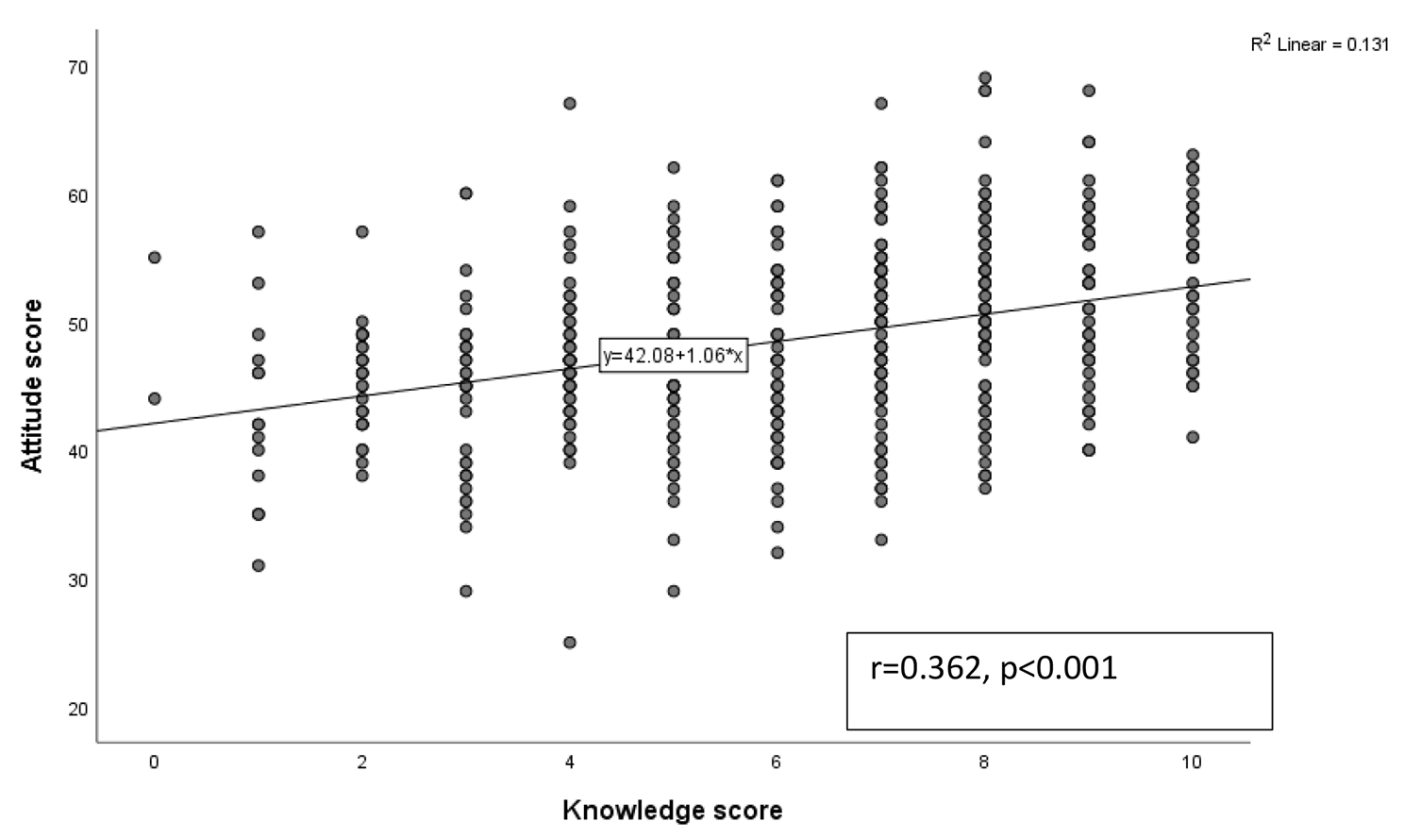
Correlation between medical students’ level of knowledge and their level of attitude

The correlation coefficient value was 0.362, which suggests a significant positive correlation between knowledge scores and attitude scores. These findings suggest that individuals with higher levels of knowledge tend to have more positive attitudes than those with lower knowledge levels. (Fig. 2).

**Table 5:** Medical students’ level of attitude in relation to gender, academic grade, and medical program

Variables	Attitude level				X^2^	*P-value*
	Negative (*N* = 3)	Neutral (*N* = 153)	Positive (*N* = 306)	Total (*N* = 462)		
Gender						
Male	**2 (**0.9%)	**70 (**31.5%)	**150 (**67.6%)	**222 (**100%)	**0.856**	**0.652**
Female	**1 (**0.4%)	**83 (**34.6%)	**156 (**65.0%)	**240 (**100%)		
Academic grade						
Preclinical	**2 (**1.1%)	**51 (**27.4%**)**	**133 (**71.5%**)**	**186 (**100%**)**	**5.228**	**0.073**
Clinical	**1 (**0.4%)	**102 (**37.0%)	**173 (**62.7%)	**276 (**100%)		
Medical Program						
Mainstream	**3 (**0.9%**)**	**128 (**36.9%**)**	**216 (**62.2%) **90 (**78.3%)	**347 (**100%**)** **115 (**100%)	**10.323**	0.006*
CBMBP	**0 (**0.0%)	**25 (**21.7%)				

## Discussion

A lack of research knowledge and interest among undergraduate medical students is a common issue that has been reported in numerous studies. Many medical students may struggle with understanding the research process, including research design, data analysis, and dissemination of results. Additionally, there may be a lack of engagement or interest among medical students, which can lead to reduced motivation to pursue research opportunities. This lack of knowledge and interest can have negative consequences for the medical field, as research plays a critical role in advancing medical knowledge and improving patient care [14].

The present study’s findings on the knowledge and attitude levels of medical students at Tanta University provide valuable insights into the current state of research engagement among this population. The overall average score of the participants regarding their knowledge was of a moderate level of knowledge according to the scoring system of the study. The finding that almost half of the students had an acceptable level of research knowledge is encouraging, as it suggests that a substantial proportion of students have a good understanding of research methodology and its implementation. This could have positive implications for the future of medical research, as these students are likely to be better equipped to engage in research activities and contribute to the advancement of medical knowledge. A similar study conducted in Egypt among medical students at Ain Shams University from all academic grades [3], reported that students had a moderate level of knowledge toward research (mean score 43.4%). Another study conducted in Egypt among undergraduate medical students at Ain Shams University [15] reported a low level of knowledge among students from the second to the fourth year, ranging from 20 to 43.3%. A study conducted in Pakistan among undergraduate medical students [12] reported a moderate level of knowledge of health research (mean score of 49%) among Pakistani medical students. In another study conducted in Jeddah, Saudi Arabia [6], the overall average score of the participants regarding their knowledge was 57%. Similarly, research conducted on undergraduate medical students in Malaysia [16] showed that the student’s average score of knowledge related to research work was 56%. A study conducted in Croatia [17] found that medical students’ level of knowledge of medical research was low, with an average score of 27%.

The finding of this study that more than two-thirds of students had a positive attitude toward research is also noteworthy, as it suggests that medical students are generally interested and motivated to engage in research activities. This finding is consistent with previous research conducted in Jeddah, Saudi Arabia [6], which revealed that medical students had a positive attitude, with an average score of 76%. A study conducted at three Arab universities, namely, King Faisal University (Saudi Arabia), Arab Gulf University (Bahrain), and Kuwait University (Kuwait), among medical students [18] reported similar results, with an average score of 75.2% for the students’ attitudes toward medical research. However, a study conducted in Pakistan [12] reported that medical students’ attitudes toward medical research were relatively unacceptable, with an average score of 53.7%. A positive attitude toward research is an important predictor of research engagement, as it can drive students to seek out research opportunities and persist in the face of challenges [19].

The present study found that the most frequently reported barriers were lack of funding and lack of time, which suggests that these factors are major obstacles that hinder medical students from engaging in research activities. The lack of funding can hinder research implementation in several ways. First, it can limit the resources available to researchers, including access to equipment, data, and personnel. Second, it can restrict the scope of the research project, making it difficult to collect enough data to draw meaningful conclusions. Third, it can limit the dissemination of research findings, as researchers may not have the resources to publish their results in high-impact journals or present them at conferences [20]. Similarly, lack of time can be a significant barrier to research implementation [21]. Other responsibilities that limit the amount of time they can dedicate to research can lead to delays in completing research projects, difficulties in recruiting participants, and challenges in analyzing data.

The present study reported inadequate training in research methods as a prevalent barrier to research implementation. This suggests that medical students and other researchers may not have the necessary skills and knowledge to conduct research effectively. Similar to our findings, the study conducted in Jeddah, Saudi Arabia [6], reported that difficulty in time management was the most reported obstacle among the other mentioned factors. This is also similar to a study conducted in India [22] and a study conducted among postgraduate students at the Pravara Institute of Medical Sciences University of Central India [23]. reported that lack of financial support and inadequate guidance from senior faculty staff were also significant barriers hindering medical students from conducting clinical research.

Students’ involvement in research activities. This study found that a high proportion of students had undergone research training and engaged in research activities, which is consistent with previous research conducted in Sweden [24]. This finding indicates that medical students have a research interest and are willing to engage in research activities. However, our study also reported that only a small proportion of students had published research works or made research presentations, which is consistent with a previous study conducted on medical students in the United States of America [25], which found that the percentage of participants who were interested in research experience was 85%, whereas 92% had no publications.

In the current study, there was no significant difference between male and female students regarding their levels of knowledge and attitude. This is consistent with a study conducted among medical students at Ain Shams University [3]. where there was no significant impact of gender on students’ knowledge or attitudes. However, in a study conducted in Pakistan [12], gender did not impact knowledge on health research, but males scored significantly higher in terms of attitude than females. Meanwhile, a study conducted in Saudi Arabia [26] reported that female students had more positive attitudes toward research than male students.

The current study found that CBMBP students had significantly higher levels of knowledge and attitudes than mainstream students. This finding suggests that there may be differences in the research training and education provided by the CBMBP program compared to the mainstream program. Further research is needed to explore these differences in more detail. Future studies could examine the specific research training and education provided by the CBMBP program and how it differs from the mainstream.

The current study examined the relationship between knowledge level and attitude toward research among medical students. A significant correlation was found, as students with an acceptable level of knowledge are more likely to have a positive attitude toward research. This finding indicates that research training and education may play a crucial role in shaping the attitudes of medical students toward research. When students have a good understanding of research methods and techniques, they may appreciate the importance of research in advancing medical knowledge and improving patient outcomes. This may lead to a positive attitude toward research and a willingness to engage in research activities [27]. Conversely, students who lack knowledge of research methods and techniques may view research as a challenging and tedious task, leading to a negative attitude toward research [28]. Further research is needed to explore the relationship between knowledge level and attitude toward research among medical students in more detail.

### Limitations of the study

Several limitations to this study should be taken into consideration when interpreting the results. First, the study was conducted at a single institution, which limits the generalizability of the findings to other institutions or contexts. Second, the study relied on self-reported data, which may be subject to bias or inaccuracies. Finally, the study did not explore the perspectives and experiences of faculty members or other stakeholders in the research process, which could provide additional insights into the challenges and opportunities for research among medical students.

### Recommendations

Based on the findings of this study, several recommendations can be made to improve the research culture and opportunities for medical students at Tanta University. First, efforts should be made to increase research training and education opportunities for medical students, with a particular focus on addressing the barriers identified in this study. This could include the development of research training programs, workshops, and seminars, as well as the provision of financial support for research activities. Second, efforts should be made to increase the visibility and dissemination of research work by medical students, with a particular focus on supporting students in publishing and presenting their research work. This could include the provision of mentorship and support for students in writing and submitting research manuscripts.

## Conclusion

Overall, medical students at Tanta University had a moderate level of knowledge about concepts of research, and their attitudes regarding research implementation were variable. Students reported different barriers such as lack of training courses, lack of time, and lack of adequate training in research methods as major barriers that prevent them from conducting research. Students with an acceptable level of knowledge were more likely to have a positive attitude toward research.

### Electronic supplementary material

Below is the link to the electronic supplementary material.

Supplementary Material 1

## Data Availability

Data are available upon reasonable request from the corresponding author.
