# Supplementary material for: Medical students’ perceptions, experiences, and barriers towards research implementation at the faculty of medicine, Tanta university
Source: BMC Med Educ. 2023 Nov 27;23:902. doi: 10.1186/s12909-023-04884-z (PMC10683156; doi:10.1186/s12909-023-04884-z)
Supplement: Supplementary file 1 — Supplementary Material 1 [file 12909_2023_4884_MOESM1_ESM.docx]

**Medical students' perceptions, experiences, and barriers towards research implementation at Faculty of Medicine, Tanta University**

| We are your colleagues in Year 6 of Faculty of Medicine Tanta University. We are conducting a research project about Medical students' perceptions, experiences, and barriers towards research.  **Please note** that any information you provide through this questionnaire will be private and confidential. Participation in this survey is completely voluntary. If you agree to participate in this study, please fill in the survey, if not feel free to return the paper to the data collectors. |
| --- |

| **Age:** | **Gender:**  Male  Female |
| --- | --- |
| **Academic year:**  **2^nd^  3^rd^  4^th^  5^th^  6^th^** | |
| **Residence:**  Urban  Rural | |
| **Medical program:** :  CBMBP Mainstream | |

| **Choose the best correct answer from the following question:** |
| --- |
| 1. **The role of a research supervisor is to?**  \| 1. Give you a reading list \| 1. Negotiate access to the research setting on the student's behalf \| \| --- \| --- \| \| 1. Provide academic support, guidance, and critical feedback on your work \| 1. Ensure you keep to your schedule and deadlines \|  1. **Which of the following is not a characteristic of a research?**  \| 1. Research is systematic \| 1. Research is problem oriented \| \| --- \| --- \| \| 1. Research is not a process \| 1. Research is not passive \|  1. **Research is?**  \| 1. A lab experiment \| 1. A systemic enquiry \| \| --- \| --- \| \| 1. A report \| 1. A procedure \|  1. **The research is always?**  \| 1. Exploring new knowledge \| 1. Filling the gap between knowledge \| \| --- \| --- \| \| 1. Verifying the old knowledge \| 1. All of these \|  1. **Which one is called non-probability sampling?**  \| 1. Quota sampling. \| 1. Cluster sampling. \| \| --- \| --- \| \| 1. Systematic sampling. \| 1. Stratified random sampling. \|  1. **Which one is not an analytical study?**  \| 1. Case report \| 1. Cohort \| \| --- \| --- \| \| 1. Case-control \| 1. Randomized controlled trials \|  1. **Snapshot of population at a point of time is?**  \| 1. Case report \| 1. Cross-sectional \| \| --- \| --- \| \| 1. Cohort \| 1. Randomized controlled trials \|  1. **The research participant described in detail in which section of research plan?**  \| 1. Introduction \| 1. Method \| \| --- \| --- \| \| 1. Data analysis \| 1. Discussion \|  1. **Why do you need to review the existing literature?**  \| 1. To make sure you have a long list of references \| 1. To find out what is already known about your area of interest \| \| --- \| --- \| \| 1. Because without it, you could never reach the required word-count \| 1. To help in your general studying \|  1. **Which item is not a part of a scientific paper?**  \| 1. Discussion. \| 1. Introduction. \| \| --- \| --- \| \| 1. Letter to the editor. \| 1. Method. \| |

| **Mark one of the following to express to which extent you agree or disagree with these statements:** | **Strongly disagree** | **Disagree** | | **Neutral** | | **Agree** | **Strongly agree** |
| --- | --- | --- | --- | --- | --- | --- | --- |
| 1. I think I’m aware about research |  | |  |  | |  |  |
| 1. Research is mainly testing hypotheses |  | |  |  | |  |  |
| 1. Research means gathering information |  | |  |  | |  |  |
| 1. Research means appraising information |  | |  |  | |  |  |
| 1. I think medical research is a good career for me |  | |  |  | |  |  |
| 1. Research training should be a part of undergraduate curriculum |  | |  |  | |  |  |
| 1. Research should be incorporated in professional training |  | |  |  | |  |  |
| 1. Performing research is useful and valuable for my profession |  | |  |  | |  |  |
| 1. The skills acquired in research will be helpful to me in future |  | |  |  | |  |  |
| 1. Research is important to advance knowledge |  | |  |  | |  |  |
| 1. Performing research is stressful |  | |  |  | |  |  |
| 1. Performing research is a complex subject |  | |  |  | |  |  |
| 1. I feel insecure concerning the analysis of research data |  | |  |  | |  |  |
| 1. I enjoy performing research |  | |  |  | |  |  |
| 1. Most students benefit from research |  | |  |  | |  |  |
| 1. Research-orientated thinking plays an important role in everyday life |  | |  |  | |  |  |
| 1. Research is important to discover new things |  | |  |  | |  |  |
| 1. I find it difficult to understand the concepts of research |  | |  |  | |  |  |
| 1. I am worried that I will make many mistakes in research |  | |  |  | |  |  |
| **To which extent you consider these items as research barriers** | | | | | | | |
| 1. Difficulty in following up |  | |  |  | |  |  |
| 1. Difficulty in obtaining samples |  | |  |  | |  |  |
| 1. Lack of knowledge (about how to conduct research) |  | |  |  | |  |  |
| 1. Difficulty obtaining approval |  | |  |  | |  |  |
| 1. Poor accessibility to data base |  | |  |  | |  |  |
| 1. Lack of professional supervisors |  | |  |  | |  |  |
| 1. Lack of training courses |  | |  |  | |  |  |
| 1. Lack of time |  | |  |  | |  |  |
| 1. Lack of funding |  | |  |  | |  |  |
| 1. Lack of research ideas |  | |  |  | |  |  |
| 1. Can’t conduct data analysis |  | |  |  | |  |  |
| 1. No adequate training in research methods |  | |  |  | |  |  |
| **Experiences or practicing research** | | | **Yes** | | **No** | | |
| 1. Previous research training | | |  | |  | | |
| 1. Previous research involvement | | |  | |  | | |
| 1. Previous research publication | | |  | |  | | |
| 1. Previous research presentation | | |  | |  | | |
| 1. Currently working on research | | |  | |  | | |
